# Measuring Agreement Using Guessing Models and Knowledge Coefficients

**DOI:** 10.1007/s11336-023-09919-4

**Published:** 2023-06-08

**Authors:** Jonas Moss

**Affiliations:** Department of Data Science and Analytics, BI Norwegian Business School, Oslo, Norway

**Keywords:** Agreement, Interrater reliability, AC1, Cohen’s kappa

## Abstract

Several measures of agreement, such as the Perreault–Leigh coefficient, the $\textsc {AC}_{1}$, and the recent coefficient of van Oest, are based on explicit models of how judges make their ratings. To handle such measures of agreement under a common umbrella, we propose a class of models called guessing models, which contains most models of how judges make their ratings. Every guessing model have an associated measure of agreement we call the knowledge coefficient. Under certain assumptions on the guessing models, the knowledge coefficient will be equal to the multi-rater Cohen’s kappa, Fleiss’ kappa, the Brennan–Prediger coefficient, or other less-established measures of agreement. We provide several sample estimators of the knowledge coefficient, valid under varying assumptions, and their asymptotic distributions. After a sensitivity analysis and a simulation study of confidence intervals, we find that the Brennan–Prediger coefficient typically outperforms the others, with much better coverage under unfavorable circumstances.

**Supplementary Information:**

The online version contains supplementary material available at 10.1007/s11336-023-09919-4.

The most popular measures of agreement are chance-corrected. These can usually be written on the form

0.1 $$\begin{aligned} \frac{p_{a}-p_{ca}}{1-p_{ca}}, \end{aligned}$$

where $p_{a}$ is the percent agreement and $p_{ca}$ is a notion of chance agreement. The best known coefficients in this class are the (weighted) Cohen’s kappa 1960; 1968, Krippendorff’s 1970 alpha, Scott’s 1955 pi, and Fleiss’ 1971 kappa. The difference between these measures lies solely in their definition of the chance agreement, $p_{ca}$. These coefficient make few to no assumptions about the underlying distribution of ratings, and can be regarded as non-parametric.

It is also possible to model the judgment process directly, and then attempt to derive reasonable chance-corrected measures of agreement from these models (Janes, 1979). Examples of measures of agreements developed in this way include the Perreault–Leigh coefficient (Perreault & Leigh, 1989), the $\textsc {AC}_{1}$ (Gwet, 2008), Maxwell’s RE coefficient (Maxwell, 1977), Aickin’s $\alpha$ (Aickin, 1990), the estimators of Klauer and Batchelder (1996), and the more recent coefficient of van Oest (van Oest, 2019; van Oest & Girard, 2021). These measures of agreement depend on the parameters of the underlying judgment process, and may be considered semi-parametric instead of non-parametric. The models used by the above-mentioned authors may be called *guessing models*, as they represent ratings as being either known or guessed.

To make it clear what these models are about, consider the “textbook case argument” of Grove et al. (1981) (see Gwet 2014, Chapter 4, for an extended justification). When two judges classify people into, say, psychiatric categories, some people are bound to be “textbook cases”, i.e., being classifiable without much effort. Disagreement between competent judges will mostly occur when subjects are hard to classify, when the judges have to guess. But judges may agree on hard subjects as well, simply due to chance. We can then define a coefficient of “agreement due to knowledge” as the proportion of textbook cases.

The guessing model, introduced in the next section, will encompass the textbook case model and many more. As we will show, it is a generalization of several judgment process models discussed in the literature on measures of agreement. Any guessing model is associated with a *knowledge coefficient, *a measure of agreement defined directly from its parameters. These coefficients generalize the “agreement due to knowledge” from Grove’s textbook case to more general settings. The knowledge coefficient can, under various additional assumptions, be easily estimated from the data; the details are in Theorem 2. In some cases, it equals already established coefficients such as the Brennan–Prediger coefficient Brennan and Prediger (1981) or Fleiss’ kappa, but we will establish some less familiar formulas as well. We provide methods for doing inference for our proposed coefficients, based on the delta rule and the theory of *U*-statistics. Using sensitivity analyses and confidence interval simulations, we find that the Brennan–Prediger coefficient generally outperforms its competitors as an estimator of the knowledge coefficient, with reasonably small bias and variance in a variety of circumstances.

## Guessing Models

We work in the setting where one rating is definitely true, such as psychiatric diagnoses. Thus we exclude problems such as measuring agreement between movie reviewers, where there is no true rating. We also exclude measures of agreement between continuous measurement instrument, as continuous ratings are rarely exactly right. For instance, an instrument for measuring blood glucose may be decidedly better than another, but will never be precisely on the spot.

We will consider only agreement studies with a rectangular design, i.e., when *R* judges rate *n* items into one of $C<\infty$ categories, with every item being rated exactly once by every judge. Moreover, we will understand the set of judges as fixed and the set of items as being random and increasing with *n*. These assumptions may not be necessary for all of the results in this paper, but will make the presentation easier to follow, and are necessary for the asymptotic results. Denote the probability that the *R* judges will rate an item as belonging to the categories $x=(x_{1},x_{2},\ldots x_{R})$ by *p*(*x*).

The joint distribution of the *guessing model* is

1.1 $$\begin{aligned} p(x\mid s,x^{\star })=\prod _{r=1}^{R}\left[ s_{r}1[x_{r}=x^{\star }]+(1-s_{r})q_{r}(x_{r})\right] . \end{aligned}$$

where

- $x_{r}$ is the rating given by the *r*th judge on an item.
- $s=\{s_{1},s_{2},\ldots ,s_{r}\}$ are the *skill-difficulty parameters*, the probabilities that the *r*th judge knows the correct classification of an item. The skill-difficulty parameters can be deterministic or random. For instance, they can be sampled from a Beta distribution.
- $x^{\star }$ is the true classification the item, an unknown latent variable. These are assumed to be independent of the skill-difficulty parameters *s*. The distribution of $x^{\star }$ is $t(x^{\star })$, the *true rating distribution*.
- $q_{r}(x)$ are the *guessing distributions*, the distributions the ratings are drawn from when the *r*th judge does not know the true classification of the item.

We may use $t(x^{\star })$ to remove the dependence on $x_{i}^{\star }$ from the univariate guessing model, giving

1.2 $$\begin{aligned} p(x_{r}\mid s)=s_{r}t(x_{r})+(1-s_{r})q_{r}(x_{r}). \end{aligned}$$

The interpretation of $p(x_{r}\mid s)$ is straight-forward. When faced with an item, a judge *r* knows its true classification, drawn from *t*(*x*), with probability $s_{r}$. If the judge doesn’t know the true classification, the rating will be drawn at random from his potentially idiosyncratic guessing distribution $q_{r}(x)$. We do not allow for guessing distributions $q_{r}$ that depend both on both the true classification $x^{\star }$ and the judge, as it would make the parameters unidentifiable.

We have said nothing about the joint distribution of $(s_{1},s_{2},\dots ,s_{R},x^{\star })$ except that $x^{\star }$ is independent of the skill-difficulty parameters. This assumption is not realistic in all situations. For instance, correctly diagnosing patients with Down syndrome is easier than correctly diagnosing patients with ADHD, implying that $E[s_{r}\mid x^{\star }=\text {Down syndrome]}$ dominates $E[s_{r}\mid x^{\star }=\text {ADHD]}$, which violates independence of $s_{r}$ and $x^{\star }$. The independence assumption is not needed for the definition of the guessing model to make sense, but will be used in the remainder of the paper as it is required for Theorem 2.

In most settings with latent parameters one would decide on a model for them, such as multivariate normal in the case of linear random effects models. Instead of following this route, we will impose additional assumptions on the skill-difficulty parameters *s*, the guessing distributions $q_{r}$, and/or the true distribution *t* to make the problem manageable.

The guessing model 1.1 has, to our knowledge, not been presented in this generality before. Klauer and Batchelder (1996, Theorem 5 and Section 9) define a model of almost as high generality, but does not allow the the skill-difficulty parameters to differ between items.

### Knowledge Coefficient

We have introduced the guessing model in order to define the notion of “agreement due to knowledge” in a precise way. To gain an intuition about what we’re getting at, first consider the case of two judges with potentially different, but deterministic, skill-difficulty parameters $s_{1}$ and $s_{2}$. The probability that two judges agree on the classification of an item because they both know its classification is the product of their skill parameters, or $\nu =s_{1}s_{2}$. As “agree on classification of an item because they both know its classification” is cumbersome to read, we will call it “knowledgeable agreement” or “agree knowledgeably” from now. Extending this notion to *R* judges, $$\nu =\left( {\begin{array}{c}R\\ 2\end{array}}\right) ^{-1}\sum _{r_{1}>r_{2}}s_{r_{1}}s_{r_{2}}$$ is the probability that a randomly selected pair of judges will agree knowledgeably on a pair of ratings.

Another simple case happens when there are *R* judges with random skill-difficulty parameters that do not wary across judges when the item is fixed, i.e., $S_{1}=S_{2}=\cdots =S_{R}$, where we use capital letters to emphasize that the $S_{r}$ are random. Now $E(S_{r}^{2})$ is the probability of knowledgeable agreement. Finally, in the general guessing model, we find that the probability of knowledgeable agreement among two judges is

1.3 $$\begin{aligned} \nu =\left( {\begin{array}{c}R\\ 2\end{array}}\right) ^{-1}\sum _{r_{1}>r_{2}}E[S_{r_{1}}S_{r_{2}}]. \end{aligned}$$

### Earlier Guessing Models

The guessing model and its associated knowledge coefficient are extensions, formalizations, or slight modifications of models or coefficients used in several earlier papers.

#### The Two Models of Maxwell (1977)

Maxwell (1977, Section 3) works in the setting of two judges and binary ratings. From his Table II one can derive the the joint model for two ratings $x_{1},x_{2}$ by two judges as

1.4 $$\begin{aligned} p(x_{1},x_{2})=\alpha p(x_{1})1[x_{1}=x_{2}]+(1-\alpha )p(x_{1})p(x_{2}), \end{aligned}$$

where *p* is the marginal distribution of the data. Maxwell then shows that $\alpha ={{\,\textrm{Cor}\,}}(X_{1},X_{2})$.

Maxwell’s joint distribution is the unconditional variant of a guessing model (1.1) with two judges, i.e., a model on the form

$$\begin{aligned} p(x_{1},x_{2}\mid s,x^{\star })&=\{s_{1}1[x_{1}=x^{\star }]+(1-s_{1})q_{r}(x_{1})\}\{s_{2}1[x_{2}=x^{\star }]+(1-s_{2})q_{r}(x_{2})\} \end{aligned}$$

with associated knowledge coefficient $\nu =\alpha$. The guessing model satisfies

(i) The judges’ guessing distributions are equal to the marginal distribution, i.e., $q_{1}(x)=q_{2}(x)=p(x)$.
(ii) The true distribution is assumed to be equal to the marginal distribution, i.e., $t(x)=p(x)$.
(iii) Both judges share the same skill-difficulty parameter *s*. It is Bernoulli distributed with success probability $\alpha$, so that $\alpha =P(s=1)=Es^{2}$. Then *s* will 1 if the the case is easy to judge (i.e., a textbook case) and 0 otherwise, and the probability of an item being a textbook case is $\alpha$.

In Section 4, Maxwell (1977) is still working with binary data and two judges. He describes a guessing model where (iii) above still holds, but (i) and (ii) are replaced with

(i) Both the judges’ guessing distributions are uniform, i.e., $q_{1}(x)=q_{2}(x)=1/2$ and
(ii) The true distribution *t*(*x*) is arbitrary.

Then he derives the knowledge for this model, the Maxwell RE (abbreviation of *random error*) coefficient for binary data, a special case of the Brennan–Prediger coefficient,

1.5 $$\begin{aligned} \nu _{BP}=\frac{p_{a}-1/C}{1-1/C}, \end{aligned}$$

where *C*, in this case equal to 2, is the number of categories.

#### Perreault–Leigh Coefficient (1989)

Perreault and Leigh (1989) devise an explicit model for the rating procedure involving two judges and $C<\infty$ categories. Using an index for reliability $s\in [0,1]$, they define the univariate model

1.6 $$\begin{aligned} p(x)=st(x)+(1-s)C^{-1}. \end{aligned}$$

The model is similar to the second Maxwell model, except that the skill-difficulty parameters are deterministic and constant across judges and the number of categories is arbitrary. The guessing distributions are uniform, $q_{1}=q_{2}=1/C$, and *t*(*x*) is arbitrary. From this model they derive that $s=\sqrt{(p_{a}-1/C)/(1-1/C)}=\sqrt{\nu _{BP}}$, the square root of the Brennan–Prediger coefficient. Hence the knowledge coefficient is $\nu =s^{2}.$

#### Aickin’s Coefficient (1990)

Aickin (1990) works in a setting of two judges. He defines the joint model for two ratings $x_{1},x_{2}$ by two judges as

1.7 $$\begin{aligned} p(x_{1},x_{2})=(1-\alpha )q_{1}(x_{1})q_{2}(x_{2})+\alpha 1[x_{1}=x_{2}]\frac{q_{1}(x_{1})q_{2}(x_{1})}{\sum _{x=1}^{C}q_{1}(x_{1})q_{2}(x_{1})}, \end{aligned}$$

with the goal of doing inference on $\alpha$. He does this using maximum likelihood, estimating the distributions $q_{1}$ and $q_{2}$ alongside $\alpha$.

Aickin’s model is a guessing model and $\alpha =\nu$ is its knowledge coefficient. The assumptions of the guessing model are:

(i) As in the first Maxwell model, both judges share the same skill-difficulty parameter *s*. It is Bernoulli distributed with success probability $\alpha$, so that $\alpha =P(s=1)=Es^{2}$.
(ii) The judges’ guessing distributions are arbitrary.
(iii) The true distribution is assumed to be equal to $t(x)=q_{1}(x)q_{2}(x)/\sum _{x=1}^{C}q_{1}(x)q_{2}(x)$.

That Aicken’s model is a guessing model satisfying conditions (i)–(iii) is a direct consequence of the following fact. Whenever the number of judges is two and the skill-difficulty parameter $s\sim \text {Bernoulli}(\alpha )$ is the same for both judges, the guessing model has unconditional distribution

1.8 $$\begin{aligned} p(x_{1},x_{2})=\alpha 1[x_{1}=x_{2}]t(x_{1})+(1-\alpha )q_{1}(x_{1})q_{2}(x_{2}). \end{aligned}$$

The details are in the appendix, p. 22.

Assumption (iii) is not justified by Aickin, and does not appear to be necessary. If we define the generalized Aicken model as the guessing model satisfying only (i) and (ii) above, with an arbitrary number of judges $R\ge 2$, its parameters $(\nu ,q_{1},q_{2},...q_{R})$ are identified when $C>2$. This can be shown following the arguments laid out in the proof of Theorem 1 of Klauer and Batchelder (1996).

#### The Klauer–Batchelder Model (1996)

Klauer and Batchelder (1996) performs a detailed structural analysis of the guessing model with identical skill-difficulty parameters. In our notation, their equation 2 is

1.9 $$\begin{aligned} p(x\mid s,x^{\star })=s1[x=x^{\star }]+(1-s)q_{r}(x), \end{aligned}$$

where the guessing distributions $q_{1},q_{2}$ and the true distribution *t* are arbitrary. They show that, provided the number of judges is equal to two, then (Klauer & Batchelder 1996, eq. 3)

1.10 $$\begin{aligned} p(x_{1},x_{2})=p(x_{1})p(x_{2})+s^{2}t(x_{1})[1[x_{1}=x_{2}]-t(x_{2})], \end{aligned}$$

which does not depend directly on the guessing distributions $q_{r}(x)$. Equation (1.10) provides a nice interpretation of the skill-difficulty parameter *s*: The higher *s* is, the more weight will be on the main diagonal of the agreement matrix and less on the off-diagonal elements. Moreover, in Theorem 5, they extend equation 1.10 to the case of two judges with different deterministic skill-difficulty parameters.

They show the model is identified when the $C>2$, and propose to estimate it by maximum likelihood using the EM algorithm developed by Hu and Batchelder (1994). In addition, they show that $s^{2}$ equals Cohen’s kappa when both guessing distributions are equal to the true distribution; they also show that $s^{2}$ equals the Brennan–Prediger coefficient when both distributions are uniform. We generalize these results to arbitrary skill-difficulty parameters and an arbitrary number of judges in Theorem 2 below.

#### Van Oest’s Coefficient (2019)

Modifying the setup of Perreault and Leigh (1989), van Oest (2019) develops a guessing model for two judges and $C<\infty$ categories. He assumes the guessing distributions are equal to the marginal distribution, i.e., $q_{1}(x)=q_{2}(x)=p(x)$, and that the skill-difficulty coefficients are deterministic and constant across judges. The marginal model becomes

1.11 $$\begin{aligned} p(x)=st(x)+(1-s)p(x). \end{aligned}$$

van Oest (2019) proceeds to show that *s* equals the weighted Scott’s pi under these circumstances.

## The Knowledge Coefficient

### Definitions

Let *w*(*x*, *y*) be an* agreement weighting function*. This is a function of two arguments that satisfies $w(x,y)\le 1$ and equals 1 when $x=y$, i.e., $w(x,x)=1$. The purpose of this function is to measure the degree of similarity between *x* and *y*, where 1 is understood as the maximal degree of similarity. While there are infinitely many weighting functions, only three are in widespread use. The first is the *nominal weight*,

$$\begin{aligned} w(x_{1},x_{2})=1[x_{1}=x_{2}]={\left\{ \begin{array}{ll} 1, &{} x_{1}=x_{2},\\ 0, &{} \text {otherwise}. \end{array}\right. } \end{aligned}$$

With this function, similarity does not come in degrees, but it does with the *quadratic weighting function*, $w(x_{1},x_{2})=1-(x_{1}-x_{2})^{2}$. The *absolute value weighting function* (sometimes called the *linear* weighting function) measures the similarity between *x*, *y* using the absolute value, i.e., $w(x_{1},x_{2})=1-|x_{1}-x_{2}|$.

#### Definition 1

Recall that $p_{r}(x)$ is the marginal distribution of ratings for judge *r*, and let $x_{1}$ and $x_{2}$ be ratings by two different judges $r_{1}$ and $r_{2}$. Define the *weighted agreement* as

2.1 $$\begin{aligned} p_{wa}= & {} \left( {\begin{array}{c}R\\ 2\end{array}}\right) ^{-1}\sum _{r_{1}>r_{2}}\sum _{x_{1},x_{2}}w(x_{1},x_{2})p(x_{r_{1}},x_{r_{2}}), \end{aligned}$$

the *weighted Cohen-type chance agreement* as

2.2 $$\begin{aligned} p_{wc}= & {} \left( {\begin{array}{c}R\\ 2\end{array}}\right) ^{-1}\sum _{r_{1}>r_{2}}\sum _{x_{1},x_{2}}w(x_{1},x_{2})p(x_{r_{1}})p(x_{r_{2}}), \end{aligned}$$

and the *weighted Fleiss-type chance agreement *as

2.3 $$\begin{aligned} p_{wf}= & {} R^{-2}\sum _{r_{1},r_{2}}\sum _{x_{1},x_{2}}w(x_{1},x_{2})p(x_{r_{1}})p(x_{r_{2}}). \end{aligned}$$

The difference between the two notions of weighted chance agreement should be clear enough. The Fleiss-type probability of chance agreement counts the cases when a judge agrees with himself, while the Cohen-type does not.

Letting $\{x_{1},x_{2},\ldots ,x_{C}\}$ be the set of possible ratings and *w* an agreement weighting function, define the weighting matrix *W* as the $C\times C$ matrix with elements $W_{ir}=w(x_{i},x_{r})$. Using *W*, we can write $p_{wf}=p^{T}Wp,$ where $p=R^{-1}\sum _{r}p_{r}$ is the marginal distribution of ratings, and $$p_{wc}=\left( {\begin{array}{c}R\\ 2\end{array}}\right) ^{-1}\sum _{r_{1}>r_{2}}p_{r_{1}}^{T}Wp_{r_{2}}$$.

When *w* is the nominal weight, $p_{wa},p_{wc}$, and $p_{wf}$ are proper probabilities, and are often referred to as unweighted probabilities of (chance) agreement. We do not require that the weighting functions to be non-negative, hence the quantities $p_{wa},p_{wc}$, and $p_{wf}$ are not, in general, proper probabilities. Since the number of categories *C* is finite, however, we may assume that the weighting function is positive by normalizing, i.e., redefining the weighting function to $1-[1-w(x_{1},x_{2})]/\max _{x_{1},x_{2}}(1-w(x_{1},x_{2}))$.

### The Knowledge Coefficient Theorem

As we have seen, well-known coefficients such as Scott’s pi and the Brennan–Prediger can be understood as knowledge coefficients, albeit under restrictive assumptions such as all judges being equally skilled. The following theorem describes less stringent sets of assumptions that keeps interpretable expressions for the knowledge coefficient. We must assume either that all guessing distributions are equal or that the skill-difficulty parameters have zero pairwise covariance to get anywhere. In addition, we must assume something about either the true distribution or the guessing distribution. We never have to assume that every judge is equally competent, and we never have to assume anything about the number of categories rated, however. The content of the theorem, including the required assumptions, is summarized in Table 1.

**Table 1:** Coefficients covered in this paper.

**Assumptions**	**Name**	**Definition**
**Provided that all guessing distributions are equal**		
Guessing $=$ Uniform	Brennan–Prediger coefficient	$\nu _{BP}=\frac{p_{a}-1/C}{1-1/C}$
Guessing $=$ Marginal$^{*}$ or	Weighted Fleiss’ kappa	$\nu _{F}=\frac{p_{wa}-p_{wf}}{1-p_{wf}}$
Guessing $=$ True$^{*}$ or	Weighted Cohen’s kappa	$\nu _{C}=\frac{p_{wa}-p_{wc}}{1-p_{wc}}$
True $=$ Marginal$^{*}$	*Cohen–Fleiss coefficient*	$\nu _{CF}=\frac{p_{wa}-p_{wc}}{1-p_{wf}}$
**Provided ** $E[s_{r_{1}}s_{r_{2}}]=E[s_{r_{1}}]E[s_{r_{2}}]$ for all $r_{1},r_{2}$		
True $=$ Marginal	*Cohen–Fleiss coefficient*	$\nu _{CF}=\frac{p_{wa}-p_{wc}}{1-p_{wf}}$
True $=$ Uniform	*Cohen–Brennan–Prediger coefficient*	$\nu _{CBP}=\frac{p_{wa}-p_{wc}}{1-{\varvec{1}}^{T}W{\varvec{1}}/C^{2}}$

Note: New coefficients in italics

$^{*}$These three conditions are equivalent, see (i) of Theorem 2

#### Theorem 2

(Knowledge Coefficient Theorem) Let *w* be any agreement weighting function and *W* its associated agreement weighting matrix. Then the following holds:

(i) Assume all guessing distributions are equal, i.e, $q_{r}(x)=q(x)$. Then the following are equivalent:
  $$\begin{aligned} q(x)=t(x),\quad p(x)=t(x),\quad q(x)=p(x) \end{aligned}$$
  Assuming either of these,$p_{wc}=p_{wf}$, and the knowledge coefficient equals both the weighted multi-rater Cohen’s kappa (Conger’s kappa) and the weighted Fleiss’ kappa
  $$\begin{aligned} \nu =\nu _{C}=\frac{p_{wa}-p_{wc}}{1-p_{wc}};\quad \nu =\nu _{F}=\frac{p_{wa}-p_{wf}}{1-p_{wf}}. \end{aligned}$$
(ii) Assume all guessing distributions $q_{r}$ are uniform. Then the knowledge coefficient equals the Brennan–Prediger coefficient,
  $$\begin{aligned} \nu =\nu _{BP}=\frac{p_{a}-1/C}{1-1/C}, \end{aligned}$$
  where $p_{a}$ denotes the weighted agreement with nominal weights.
(iii) Assume that the skill-difficulty parameters have pairwise covariance equal to zero, i.e., $E[s_{r_{1}}s_{r_{2}}]=E[s_{r_{1}}]E[s_{r_{2}}]$ for all $r_{1},r_{2}$. Then the knowledge coefficient equals
  $$\begin{aligned} \nu =\frac{p_{wa}-p{}_{wc}}{1-t^{T}Wt}. \end{aligned}$$
  In particular, if $t(x)=p(x)$, it equals the “Cohen–Fleiss” coefficient
  $$\begin{aligned} \nu =\nu _{CF}=\frac{p_{wa}-p_{wc}}{1-p_{wf}}. \end{aligned}$$
  Moreover, if *t* is uniform, it equals the “Cohen–Brennan–Prediger” coefficient,
  $$\begin{aligned} \nu =\nu _{CBP}=\frac{p_{wa}-p_{wc}}{1-{\varvec{1}}^{T}W{\varvec{1}}/C^{2}}. \end{aligned}$$

#### Proof

The knowledge coefficient theorem is proved in the appendix, page 18. $\square$

Every coefficient in the theorem can be estimated by substituting the values of $p_{wa}$, $p_{wf}$, and $p_{wc}$ for their sample variants. Under any of the equivalent conditions of Theorem 2 part (i), we have that that $\nu$ equals

$$\begin{aligned} \nu _{F}=\frac{p_{wa}-p_{wf}}{1-p_{wf}},\quad \nu _{C}=\frac{p_{wa}-p_{wc}}{1-p_{wc}},\quad \nu _{CF}=\frac{p_{wa}-p_{wc}}{1-p_{wf}}. \end{aligned}$$

The coefficient $\nu _{F}=\frac{p_{wa}-p_{wf}}{1-p_{wf}}$ is a weighted Fleiss’ kappa. This coefficient is strongly related to the weighted Krippendorff’s alpha, which we denote by ${\hat{\alpha }}$. Indeed, it is easy to see that ${\hat{\alpha }}$ is a linear transformation of ${\hat{\nu }}_{F}$,

2.4 $$\begin{aligned} {\hat{\alpha }}={\hat{\nu }}_{F}+\frac{1}{N}(1-{\hat{\nu }}_{F}), \end{aligned}$$

where *N* is the total number of ratings made (see the appendix of, Moss, J (2023)) and ${\hat{\nu }}_{F}$, the sample weighted Fleiss kappa. Thus ${\hat{\alpha }}$ is a consistent estimator of $\nu _{F}$.

On the other hand, $\nu _{C}$ is a weighted multi-rater Cohen’s kappa of the form discussed by Berry, K& J., Mielke, P. W. (1988) and Janson and Olsson (2001); it can also be regarded as a weighted Conger’s kappa (Conger, 1980). I have not seen anything like $\nu _{CF}$, a curious combination of Cohen’s kappa and Fleiss’ kappa, probably because it is not a classical chance-corrected chance measure of agreement, as its numerator chance agreement $p_{wc}$ is distinct from the denominator chance agreement $p_{wf}$. The required condition for the Cohen–Fleiss coefficient, $p(x)=t(x)$, holds if and only if

$$\begin{aligned} R^{-1}\sum _{r=1}^{R}\left[ (1-s_{r})q_{r}(x)+s_{r}t(x)\right] =t(x)\Longleftrightarrow \sum _{r=1}^{R}(1-s_{r})q_{r}(x)=t(x)\sum _{r=1}^{R}(1-s_{r}),\quad \text {for all }r. \end{aligned}$$

which hols trivially if $q_{r}(x)=t(x)$ for all *r*. Hence the Cohen–Fleiss coefficient is consistent for the population knowledge coefficient under strictly more situations than weighted Fleiss’ kappa and weighted multi-rater Cohen’s kappa, and may be preferred to them by a researcher who buys the rationale behind the guessing model.

The Cohen-Brennan–Prediger coefficient will be equal to the knowledge coefficient if the true distribution equals the uniform distribution and the skill-difficulty parameters have pairwise covariances equal to zero. This scenario may be uncommon, but can happen if the designer of the agreement study has complete control over the true ratings.

Part (ii) of Theorem 2 concerns the case when a judge who does not know the correct classification of an item guesses uniformly at random, so that $q_{r}(x)=C^{-1}$ for all judges *r*. This assumption is used by e.g. Brennan and Prediger (1981), Maxwell (1977), Gwet (2008) and Perreault and Leigh (1989).

#### Example 3

Zapf et al. (2016) did a case study on histopathological assessment of breast cancer. The number of judges was $R=4$, the number breast cancer biopsies rated was $n=50,$and the number of categories $C=5$. The estimated coefficients are

$$\begin{aligned} {\hat{\nu }}_{CF}=0.574,\quad {\hat{\nu }}_{F}=0.562,\quad {\hat{\nu }}_{C}=0.567,\quad {\hat{\nu }}_{BP}=0.604,\quad {\hat{\nu }}_{CBP}=0.519. \end{aligned}$$

In this case, the coefficients are quite close, suggesting that the guessing distributions are close to the marginal distribution and that the marginal distribution is close to the uniform distribution. The observed marginal distribution in this data set 0.255, 0.025, 0.12, 0.21, 0.39. This appears to be quite far away from the uniform distribution, which raises the question of how sensitive the Brennan–Prediger coefficient is to the uniformity assumption.

## Sensitivity and Performance

Recall the assumptions on the coefficients of Theorem 2

- **Brennan–Prediger**: All guessing distributions are equal to the uniform distribution.
- **Cohen’s kappa, Fleiss’ kappa**: All guessing distributions are equal, the true distribution equals the marginal distribution.
- **Cohen–Fleiss**: The true distribution equals the marginal guessing distribution, $E[s_{r_{1}}s_{r_{2}}]=E[s_{r_{1}}]E[s_{r_{2}}]$ for all $r_{1},r_{2}$.
- **Cohen–Brennan–Prediger**: The true distribution is uniform, $E[s_{r_{1}}s_{r_{2}}]=E[s_{r_{1}}]E[s_{r_{2}}]$ for all $r_{1},r_{2}$.

These assumptions are quite stringent, and will realistically never hold exactly. In this section, we do two sensitivity–performance studies to check how well the coefficients perform when the assumptions are broken. Theorem 2 contains two classes of coefficients. The first class, containing the Brennan–Prediger coefficient in addition to Cohen’s and Fleiss’ kappa, requires at minimum that all guessing distributions are equal. The second class, containing the Cohen–Fleiss and Cohen–Brennan–Prediger coefficient, requires that all pairs of skill-difficulty parameters have zero covariance. We will do two studies, one where $s_{r_{1}}$ and $s_{r_{2}}$ have zero covariance and one where $s_{r_{1}}$ and $s_{r_{2}}$ are correlated. We restrict our study the the case of nominal weights.

### When $E[s_{r_{1}}s_{r_{2}}]=E[s_{r_{1}}]E[s_{r_{2}}]$

We will use the a special case of the guessing model we call the *judge skill model*. In this model the skill-difficulty parameter *s* is deterministic. This is a generalization of the models used by e.g. Perreault and Leigh (1989) and van Oest (2019) to allow for judges with different skill levels. Since *s* is deterministic, $E[s_{r_{1}}s_{r_{2}}]=E[s_{r_{1}}]E[s_{r_{2}}]$ and the main condition of Theorem 2 part (iii) is satisfied. Under the judge skill model, it is fairly easy to calculate the theoretical values of the five coefficients in Theorem 2. To calculate $p_{wa}$, we use representation (i) of Lemma 7 (p. 18), in the appendix. Since $p_{wf}=p^{T}Wp$, where *p* is the marginal distribution, and $$p_{wc}=\left( {\begin{array}{c}R\\ 2\end{array}}\right) ^{-1}\sum _{r_{1}>r_{2}}p_{r_{1}}^{T}Wp_{r_{2}}$$, the values of $p_{wf}$ and $p_{wc}$ are easily calculated.

The parameters *R*, *C* and *s* are sampled as follows:

(i) The number of judges (*R*) is sampled uniformly from [2, 20].
(ii) The number of categories (*C*) is sampled uniformly from [2, 10].
(iii) The *R* skill–difficulty parameters $s_{1},\ldots ,s_{R}$ are drawn independently from a beta distribution with parameters 7 and 1.5. This is a slightly dispersed, asymmetric distribution with a mean of 0.82.

We study what happens when the true distribution deviates from the uniform (an assumption for $\nu _{CBP}$) and / or the guessing distribution deviates from the uniform distribution (an assumption for $\nu _{BP}$). The numbers are the simulated mean absolute deviations from the true knowledge coefficient $E|\nu -\nu _{x}|$, where *x* is one of *F*, *C*, *BP*, *CF*, or *CBP*. The smallest numbers by orders of magnitude on each row is in bold.

#### True Distribution Centered on the Uniform Distribution

If the variability of the true distribution is “None”, it equals the uniform distribution. If the variability is “Low”, it is sampled from a symmetric Dirichlet distribution ( Johnson, Kotz, & Balakrishnan^1994^, Chapter 49) with concentration parameter $\alpha =10$; if variability is “High”, $\alpha =0.5$. Likewise, for the guessing distributions, if the variability is “None”, all guessing distributions are equal to the uniform distribution. If the variability is “Low”, the guessing distributions are sampled from a symmetric Dirichlet distribution with concentration parameter $\alpha =10$. Finally, if the variability is “High”, they are sampled from a symmetric Dirichlet distribution with $\alpha =0.5$.

**Table 2:** Sensitivity analysis when $E[s_{r_{1}}s_{r_{2}}]=E[s_{r_{1}}]E[s_{r_{2}}]$. True distribution centered on the uniform distribution.

	Variability			Coefficient				
	Guessing$^{\star }$	True$^{*}$		Cohen-Fleiss	Fleiss	Cohen	BP	Cohen–BP
	None	None		**0.0e+00**	**0.0e+00**	**0.0e+00**	**0.0e+00**	**0.0e+00**
		Low		3.8e-03	3.8e-03	3.8e-03	**0.0e+00**	1.2e-02
		High		7.5e-02	7.5e-02	7.5e-02	**0.0e+00**	1.7e-01
	Low	None		**5.2e-05**	**4.9e-05**	**4.3e-05**	**5.5e-05**	**1.7e-05**
		Low		3.8e-03	3.8e-03	3.8e-03	**5.7e-04**	1.2e-02
		High		7.4e-02	7.4e-02	7.4e-02	**2.0e-03**	1.7e-01
	High	None		**7.3e-04**	**8.0e-04**	**6.9e-04**	**8.7e-04**	**2.7e-04**
		Low		**3.5e-03**	**4.2e-03**	**3.9e-03**	**2.4e-03**	1.2e-02
		High		7.3e-02	7.4e-02	7.4e-02	**7.9e-03**	1.7e-01

$^{*}$Variability of the true distributions: Baseline: True distribution is uniform.

$^{\star }$Variability of the guessing distributions. Baseline: All guessing distributions are equal to the true distribution.

From Table 2 we see that the Cohen–Brennan–Prediger coefficient performs worst in every setting,, usually by a large margin, except when the true distribution is uniform. Moreover, the Brennan–Prediger coefficient performs well in every scenario. The bias $|\nu _{BP}-\nu |$ will be likely be overshadowed by sampling variability for most conceivable sample sizes. Finally, there is little difference between Cohen’s kappa, Fleiss’ kappa, and the Cohen–Fleiss coefficient. Their biases are quite small, at least when the true distribution isn’t far away from the uniform distribution.

#### True Distribution Centered on the Marginal Distribution

To derive Fleiss’ kappa, Cohen’s kappa, and the Cohen–Fleiss coefficient, we assumed that the true distribution equals the marginal distribution. We can use an asymmetric Dirichlet distribution to extend this scenario, just as we used the symmetric Dirichlet distribution to extend the scenario when the true distribution is uniform. This time we won’t use the uniform distribution as a base true distribution, but randomly generated distribution *h*, from a symmetric Dirichlet distribution with $\alpha =5$, instead. The rest of the settings are identical to the previous sensitivity study.

**Table 3:** Sensitivity analysis when $E[s_{r_{1}}s_{r_{2}}]=E[s_{r_{1}}]E[s_{r_{2}}]$. True distribution centered on the marginal guessing distribution.

	Variability			Coefficient				
	Guessing$^{\star }$	True$^{*}$		Cohen-Fleiss	Fleiss	Cohen	BP	Cohen–BP
	None	None		**0.0e+00**	**0.0e+00**	**0.0e+00**	1.1e-02	2.5e-02
		Low		**2.1e-02**	**2.1e-02**	**2.1e-02**	**1.1e-02**	**8.2e-02**
		High		3.4e-01	3.4e-01	3.4e-01	**1.8e-02**	4.7e-01
	Low	None		**1.4e-04**	**4.6e-04**	**3.6e-04**	1.3e-02	3.0e-02
		Low		**2.2e-02**	**2.2e-02**	**2.2e-02**	**1.4e-02**	**9.1e-02**
		High		3.2e-01	3.3e-01	3.3e-01	**1.8e-02**	4.6e-01
	High	None		**1.1e-03**	**3.5e-03**	**2.8e-03**	3.0e-02	7.4e-02
		Low		**2.5e-02**	**2.7e-02**	**2.6e-02**	**3.0e-02**	1.3e-01
		High		3.2e-01	3.2e-01	3.2e-01	**3.4e-02**	4.7e-01

$^{*}$Variability of the true distributions: Baseline: True distribution equals the marginal guessing distribution.

$^{\star }$Variability of the guessing distributions. Baseline: All guessing distributions are equal to the marginal guessing distribution.

The results are in Table 3. We see that the Cohen–Brennan–Prediger coefficient performs worst in every setting, usually by a large margin. Surprisingly, the Brennan–Prediger coefficient performs best in 6/9 cases, and its performance is good in the remaining cases too. Some of the biases in the table are unacceptably large. Only the Brennan–Prediger coefficient has a bias less than 0.1 in every case. Finally, there is little difference between Cohen’s kappa, Fleiss’ kappa, and the Cohen–Fleiss coefficient. The Cohen–Fleiss coefficient does better, especially when the true distribution equals the marginal distribution, but the difference in performance is insignificant under slight deviations from equality.

### When $E[s_{r_{1}}s_{r_{2}}]\ne E[s_{r_{1}}]E[s_{r_{2}}]$

To model dependent skill-difficulty parameters, let *F* be a *R*-variate distribution with $s\sim F$. Evidently, the only restriction on *F* is that it’s a multivariate distribution function on [0, 1]. A natural way to model situation is to use copulas for the dependence structure and a density on [0, 1] for the marginals (Nelsen, 2007). We will use the *R*-variate Gaussian copula with uniform correlation structure, i.e., the correlation matrix parameter

$$\begin{aligned} \left[ \begin{array}{cccc} 1 &{} \rho &{} \cdots &{} \rho \\ \rho &{} 1 &{} \cdots &{} \rho \\ \vdots &{} \vdots &{} \ddots &{} \vdots \\ \rho &{} \rho &{} \cdots &{} 1 \end{array}\right] . \end{aligned}$$

Denote this Gaussian copula by $C_{\rho }$. Let $H_{(a,b)}$ be the cumulative distribution function of a beta distribution with parameters *a* and *b*, and define

$$\begin{aligned} F_{\rho ,a,b}(s_{1},s_{2},\ldots ,s_{R})=C_{\rho }(H_{(a,b)}(s_{1}),H_{(a,b)}(s_{2}),\ldots ,H_{(a,b)}(s_{R})). \end{aligned}$$

Then $F_{\rho ,a,b}$ is a reasonable model for dependent skill-difficulty parameters.

We use the small correlation parameter $\rho =0.2$. The rest of the settings are exactly the same as the previous setting, including the beta parameters $a=7,b=1/2$. We see that the Cohen–Brennan–Prediger coefficient still outperforms the alternatives when the true distribution equals the uniform distribution, but only by a an order of magnitude. Since the alternatives to the Cohen–Brennan–Prediger coefficient also performs well in this situation, with biases likely to be overshadowed by sampling error, the case for it remains weak. Similarly, we see that the Cohen–Fleiss coefficient still outperforms Cohen’s kappa and Fleiss’ kappa, roughly by one order of magnitude, a much smaller margin than before. These comments also hold for the case of $\rho =0.5,0.9$, which can be found in the online appendix (p. 23).

**Table 4:** Sensitivity analysis when $E[s_{r_{1}}s_{r_{2}}]\ne E[s_{r_{1}}]E[s_{r_{2}}]$. True distribution centered on the uniform distribution.

	Variability			Coefficient				
	Guessing$^{\star }$	True$^{*}$		Cohen–Fleiss	Fleiss	Cohen	BP	Cohen–BP
	None	None		**0.0e+00**	**0.0e+00**	**0.0e+00**	**0.0e+00**	0.0e+00
		Low		3.8e-03	3.8e-03	3.8e-03	**0.0e+00**	1.2e-02
		High		7.4e-02	7.4e-02	7.4e-02	**0.0e+00**	1.7e-01
	Low	None		4.6e-05	4.0e-05	3.4e-05	4.5e-05	**8.7e-06**
		Low		3.5e-03	3.6e-03	3.6e-03	**5.6e-04**	1.1e-02
		High		7.8e-02	7.8e-02	7.8e-02	2.4e-03	1.7e-01
	High	None		6.3e-04	5.8e-04	4.6e-04	6.2e-04	**1.2e-04**
		Low		3.6e-03	4.3e-03	4.0e-03	**2.2e-03**	1.1e-02
		High		8.2e-02	8.4e-02	8.3e-02	**8.6e-03**	1.8e-01

$^{*}$Variability of the true distributions: Baseline: True distribution is uniform.

$^{\star }$Variability of the guessing distributions. Baseline: All guessing distributions are equal to the true distribution.

**Table 5:** Sensitivity analysis when $E[s_{r_{1}}s_{r_{2}}]\ne E[s_{r_{1}}]E[s_{r_{2}}]$. True distribution centered on the marginal guessing distribution.

	Variability			Coefficient				
	Guessing$^{\star }$	True$^{*}$		Cohen–Fleiss	Fleiss	Cohen	BP	Cohen–BP
	None	None		**0.0e+00**	**0.0e+00**	**0.0e+00**	1.0e-02	2.3e-02
		Low		2.2e-02	2.2e-02	2.2e-02	**1.2e-02**	8.4e-02
		High		3.2e-01	3.2e-01	3.2e-01	**1.8e-02**	4.5e-01
	Low	None		**7.5e-05**	3.9e-04	2.9e-04	1.3e-02	3.0e-02
		Low		**2.3e-02**	**2.3e-02**	**2.3e-02**	1.4e-02	9.1e-02
		High		3.3e-01	3.3e-01	3.3e-01	**2.1e-02**	4.6e-01
	High	None		**6.2e-04**	3.3e-03	2.4e-03	3.0e-02	7.1e-02
		Low		**2.4e-02**	2.7e-02	2.6e-02	3.1e-02	1.3e-01
		High		3.5e-01	3.5e-01	3.5e-01	**3.6e-02**	4.8e-01

$^{*}$Variability of the true distributions: Baseline: True distribution equals the marginal guessing distribution.

$^{\star }$Variability of the guessing distributions. Baseline: All guessing distributions are equal.

### Recommendations

We can draw three tentative conclusions from the sensitivity analysis.

(i) Unless the researcher can make sure the true distribution is exactly uniform, the Cohen–Brennan–Prediger coefficient is probably not worth reporting. Its bias is often unacceptably large, frequently larger than 0.1.
(ii) The Brennan–Prediger coefficient performs reasonably well in all situations, with biases less than 0.01 when the true distribution is centered on the uniform distribution. It performs decently in the second scenario as well, with biases at around 0.05 across the board.
(iii) The Cohen–Fleiss coefficient does slightly better than Cohen’s kappa and Fleiss’ kappa, but not enough to be important.

Since the Cohen–Fleiss coefficient does slightly better than Fleiss’ kappa and Cohen’s kappa, it appears prudent to report it. However, we believe it would be best not to. For both Fleiss’ kappa and Cohen’s kappa are well-known chance-corrected measures of agreement. In contrast, the Cohen–Fleiss coefficient is neither well-known nor a chance-corrected measure of agreement. We recommend that you report Cohen’s kappa (or Fleiss’ kappa) together with the Brennan–Prediger coefficient. This solution accounts both for the scenario when the marginal distribution is close to the true distribution and the scenario when the uniform distribution is close to the marginal distribution.

## Inference

The asymptotic distribution of the coefficients Table 1 can readily be calculated using the theory of *U*-statistics.

The following Lemma is instrumental in constructing the confidence intervals.

### Lemma 4

Define the parameter vectors ${\varvec{p}}=(p_{wa},p_{wc},p_{wf})$ and $\hat{{\varvec{p}}}=({\hat{p}}_{wa},{\hat{p}}_{wc},{\hat{p}}_{wf})$, and let $\Sigma$ be the covariance matrix with elements

$$\begin{aligned} \sigma _{11}&= \sigma _{A}^{2} = {{\,\textrm{Var}\,}}\mu _{wa}({\varvec{X}}_{1}) ,\quad \sigma _{12} = \sigma _{AC}^{2} = 2{{\,\textrm{Cov}\,}}\left( \mu _{wa}({\varvec{X}}_{1}),\mu _{wc}({\varvec{X}}_{1})\right) ,\\ \sigma _{22}&= \sigma _{C}^{2} = 4{{\,\textrm{Var}\,}}\mu _{wc}({\varvec{X}}_{1}) ,\quad \sigma _{13} = \sigma _{AF}^{2} = 2{{\,\textrm{Cov}\,}}\left( \mu _{wa}({\varvec{X}}_{1}),\mu _{wc}({\varvec{X}}_{1})\right) ,\\ \sigma _{33}&= \sigma _{F}^{2} = 4{{\,\textrm{Var}\,}}\mu _{wf}({\varvec{X}}_{1}) ,\quad \sigma _{23} = \sigma _{CF}^{2} = 4{{\,\textrm{Cov}\,}}\left( \mu _{wc}({\varvec{X}}_{1}),\mu _{wf}({\varvec{X}}_{1})\right) . \end{aligned}$$

Then

$$\begin{aligned} \sqrt{n}(\hat{{\varvec{p}}}-{\varvec{p}}){\mathop {\rightarrow }\limits ^{d}}N(0,\Sigma ). \end{aligned}$$

### Proof

See Lemma 1 of Moss, J (2023). The definitions of $\mu _{wa}({\varvec{X}}_{1}),\mu _{wc}({\varvec{X}}_{1})$ and $\mu _{wf}({\varvec{X}}_{1})$ can be found there. $\square$

An application of the delta method yields the following.

### Proposition 5

The coefficients in Table 1 are asymptotically normal, and their asymptotic variances are

4.1 $$\begin{aligned} \sigma _{F}^{2}= & {} \sigma _{A}^{2}\frac{1}{(1-p_{wf})^{2}}-2\sigma _{FA}\frac{1-p_{wa}}{(1-p_{wf})^{3}}+\sigma _{F}^{2}\frac{(1-p_{wa})^{2}}{(1-p_{wf})^{4}}. \end{aligned}$$

4.2 $$\begin{aligned} \sigma _{C}^{2}= & {} \sigma _{A}^{2}\frac{1}{(1-p_{wc})^{2}}-2\sigma _{CA}\frac{1-p_{wa}}{(1-p_{wc})^{3}}+\sigma _{C}^{2}\frac{(1-p_{wa})^{2}}{(1-p_{wc})^{4}}, \end{aligned}$$

4.3 $$\begin{aligned} \sigma _{BP}^{2}= & {} \sigma _{A}^{2}\frac{C^{2}}{(1-C)^{2}}, \end{aligned}$$

4.4 $$\begin{aligned} \sigma _{CF}^{2}= & {} (1-p_{wf})^{-2}\left( 1,-1,\nu _{CBP}\right) \Sigma \left( 1,-1,\nu _{CBP}\right) ^{T}, \end{aligned}$$

4.5 $$\begin{aligned} \sigma _{CBP}^{2}= & {} \frac{\sigma _{A}^{2}-2\sigma _{CA}+\sigma _{C}^{2}}{(1-{\varvec{1}}^{T}W{\varvec{1}}/C^{2})^{2}}. \end{aligned}$$

### Proof

The expressions for $\sigma _{F}^{2}$ and $\sigma _{C}^{2}$ are from Moss, J (2023, Proposition 2). The simple proof for the Cohen–Fleiss coefficient is in the appendix, p. 6. The asymptotic variance of the Brennan–Prediger coefficient is well-known and the easiest to derive. The variance of the Cohen–Brennan–Prediger coefficient follows immediately from an application of the delta method. $\square$

To estimate the $\sigma _{x}^{2}$, where *x* is a placeholder for *F*, *C*, *BP*, *CF*, or *CBP*, we use an empirical approach that coincides with that of Gwet (2021) in the special case of Fleiss’ kappa with nominal weights. See the comments following Proposition 1 of Moss, J (2023) for details.

### Confidence Intervals

Moss, J (2023) found that the arcsine interval tends to do slightly better than the untransformed interval for agreement coefficients with rectangular design. For that reason, we will only look at the arcsine interval here. Using the delta method, together with the fact that $\frac{d}{dx}\arcsin (x)=1/\sqrt{1-x^{2}}$, we find that

4.6 $$\begin{aligned} \sqrt{n}(\arcsin {\hat{\nu }}_{x}-\arcsin \nu _{x}){\mathop {\rightarrow }\limits ^{d}}N(0,(1-\nu _{x}^{2})^{-1}\sigma _{x}^{2}), \end{aligned}$$

where ${\mathop {\rightarrow }\limits ^{d}}$ denotes convergence in distribution and $\arcsin x$ is the inverse of the sine function. Again, *x* is a placeholder for *F*, *C*, *BP*, *CF*, or *CBP*. Define the arcsine interval as

4.7 $$\begin{aligned} CI_{x}=\sin \left( \arcsin {\hat{\nu }}\pm t_{1-\alpha /2}(n-1)(1-{\hat{\nu }}_{x}^{2})^{-1}{\hat{\sigma }}_{x}^{2}\right) , \end{aligned}$$

where ${\hat{\sigma }}_{x}^{2}$ is estimated empirically, as described in the previous subsection, and ${\hat{\nu }}_{x}$ is the sample estimator of $\nu _{x}$.

#### Example 6

(Example 3 (cont.)) We calculate arcsine confidence intervals along with the point estimates for the five coefficients using the data from Zapf et al. (2016). The results are in Table 6.

**Table 6:** Confidence limits for Zapf et al. (2016).

	$\nu _{CF}$	$\nu _{F}$	$\nu _{C}$	$\nu _{BP}$	$\nu _{CBP}$
Upper limit	0.68	0.67	0.67	0.70	0.62
Lower limit	0.46	0.44	0.45	0.49	0.41
Estimate	0.57	0.56	0.57	0.60	0.52

### Coverage of the Confidence Intervals

We use the arcsine interval and nominally weighted coefficients. The settings of our simulation study follows the settings of the first sensitivity analysis closely. We use $N=10,000$ repetitions and the following simulation parameters:

(i) **Number of judges** *R*. We use 2, 5, 20, corresponding to a small, medium, and large selection of judges.
(ii) **Sample sizes** *n*. We use $n=20,100$, corresponding to small and large agreement studies.
(iii) **Model.** We simulate from the judge skill model used in the sensitivity study (p. 9).

In some cases the simulation yields data frames with only identical values. Our confidence interval construction do not cover these instances, so we decided to discard these simulations, repeating the simulation until we got a data frame with at least two different values.

#### True Distribution Centered on the Uniform Distribution

We use the same setup as in 3.1.1, where we studied deviations from two assumptions. (a), that the true distribution is centered on the uniform distribution, (b) that the guessing distributions are equal.

**Table 7:** Coverage and lengths of confidence intervals, deviation from uniform.

	Coefficient	${{\,\textrm{Var}\,}}t$:	None			Low			High		
		${{\,\textrm{Var}\,}}q$:	None	Low	High	None	Low	High	None	Low	High
		$$n=20$$									
	Cohen–Fleiss		0.95	0.95	0.95	0.95	0.95	0.95	0.82	0.81	0.82
			0.26	0.26	0.26	0.26	0.26	0.26	0.33	0.33	0.33
	Fleiss		0.95	0.95	0.95	0.94	0.95	0.95	0.81	0.81	0.82
			0.26	0.26	0.26	0.27	0.27	0.27	0.33	0.33	0.34
	Cohen		0.95	0.95	0.95	0.95	0.95	0.95	0.81	0.81	0.82
			0.26	0.26	0.26	0.27	0.27	0.26	0.33	0.33	0.33
	BP		0.96	0.96	0.96	0.96	0.96	0.96	0.96	0.96	0.95
			0.26	0.26	0.26	0.26	0.26	0.26	0.26	0.26	0.26
	Cohen–BP		0.92	0.92	0.92	0.90	0.90	0.90	0.43	0.43	0.44
			0.27	0.27	0.27	0.28	0.28	0.28	0.33	0.33	0.33
		$$n=100$$									
	Cohen–Fleiss		0.95	0.95	0.95	0.94	0.94	0.94	0.52	0.52	0.53
			0.11	0.11	0.11	0.11	0.11	0.11	0.14	0.14	0.14
	Fleiss		0.95	0.95	0.95	0.94	0.94	0.93	0.51	0.52	0.51
			0.11	0.11	0.11	0.11	0.11	0.11	0.14	0.14	0.14
	Cohen		0.95	0.95	0.95	0.94	0.94	0.94	0.51	0.52	0.52
			0.11	0.11	0.11	0.11	0.11	0.11	0.14	0.14	0.14
	BP		0.95	0.95	0.95	0.95	0.95	0.95	0.95	0.95	0.90
			0.11	0.11	0.11	0.11	0.11	0.11	0.11	0.11	0.11
	Cohen–BP		0.95	0.94	0.95	0.89	0.89	0.88	0.10	0.11	0.11
			0.11	0.11	0.11	0.11	0.11	0.11	0.14	0.14	0.14

Table 7 contains the results of the simulation. All coefficients, except the Brennan–Prediger coefficient, perform poorly when *t* is far from the uniform. The Cohen–Fleiss coefficient has poor coverage when the true distribution is far away from the marginal distribution; likewise for Fleiss’ kappa and Cohen’s kappa. The Brennan–Prediger coefficient performs surprisingly well, with far better coverage than Cohen’s kappa, Fleiss’ kappa, and the Cohen–Fleiss coefficient. On the other hand, the Cohen–Brennan–Prediger coefficient performs poorly. The coverage when *t* is far from the uniform or $n=100$ is unacceptably low for all coefficients except the Brennan–Prediger coefficient.

#### True Distribution Centered on the Marginal Distribution

We use the same setup as in 3.1.2, where we studied deviations from the assumption that the true distribution is centered on the marginal distribution and that the guessing distributions are equal.

**Table 8:** Coverage and lengths of confidence intervals, deviation from marginal.

Coefficient	${{\,\textrm{Var}\,}}t$:	None			Low			High		
	${{\,\textrm{Var}\,}}q$:	None	Low	High	None	Low	High	None	Low	High
	$$n=20$$									
Cohen–Fleiss		0.95	0.95	0.95	0.93	0.92	0.92	0.37	0.37	0.39
		0.27	0.27	0.3	0.29	0.29	0.32	0.36	0.36	0.34
Fleiss		0.95	0.95	0.94	0.92	0.92	0.91	0.37	0.37	0.39
		0.27	0.28	0.31	0.30	0.30	0.34	0.36	0.37	0.36
Cohen		0.95	0.95	0.94	0.92	0.92	0.91	0.37	0.37	0.38
		0.27	0.27	0.30	0.29	0.30	0.33	0.36	0.36	0.34
BP		0.96	0.95	0.90	0.96	0.95	0.91	0.94	0.94	0.87
		0.26	0.26	0.26	0.26	0.26	0.27	0.26	0.26	0.26
Cohen–BP		0.88	0.85	0.72	0.69	0.67	0.55	0.04	0.04	0.06
		0.29	0.3	0.34	0.32	0.32	0.35	0.26	0.26	0.25
	$$n=100$$									
Cohen–Fleiss		0.95	0.95	0.95	0.85	0.85	0.84	0.09	0.09	0.13
		0.11	0.11	0.12	0.12	0.12	0.14	0.18	0.18	0.18
Fleiss		0.95	0.95	0.95	0.85	0.84	0.82	0.09	0.09	0.12
		0.11	0.11	0.13	0.12	0.12	0.14	0.18	0.18	0.19
Cohen		0.95	0.95	0.95	0.85	0.85	0.83	0.09	0.09	0.12
		0.11	0.11	0.13	0.12	0.12	0.14	0.18	0.18	0.18
BP		0.92	0.89	0.64	0.91	0.87	0.65	0.86	0.81	0.58
		0.11	0.11	0.11	0.11	0.11	0.11	0.11	0.11	0.10
Cohen–BP		0.81	0.70	0.28	0.34	0.28	0.15	0.00	0.00	0.01
		0.12	0.12	0.14	0.13	0.13	0.15	0.12	0.12	0.11

The results are in Table 8. The most striking feature is, once again, the poor performance of every coefficient except the Brennan–Prediger coefficient. The Brennan–Prediger coefficient performs well, with a coverage of approximately 0.95 in most scenarios when $n=20$. What’s more, its length is always the smallest. Cohen’s kappa, Fleiss’ kappa, and the Cohen–Fleiss coefficient performs decently, except when the marginal distribution is far away from the uniform, when their coverage is dismal. The Cohen–Brennan–Prediger coefficients performs worse than the others, with larger confidence interval lengths and horrible coverage. Compared to Table 7, the coverages in Table 8 are much worse. Even the best-performing Brennan–Prediger coefficient gets as low as 0.58 at a point.

## Concluding Remarks

In the guessing model, a judge either knows – with $100\%$ certainty – the correct classification, or he makes a guess. A more realistic model would let knowledge come in degrees. A judge could be, say, $70\%$ sure that a patient is X, $20\%$ sure that he is Y, and $10\%$ spread evenly on the remaining options. In epistemology, the *credence function* (Pettigrew, 2019) quantifies his degree of belief in the different propositions. Defining and working with knowledge coefficients in more general “credence models” might be possible, but identifiability issues looms large.

The sensitivity and coverage studies in this paper are limited in scope, as they only covers nominal weights and and a small number of parameter tweaks. Larger simulation study could potentially confirm or disconfirm our recommendation of reporting Cohen’s kappa and Fleiss’ kappa together with the Brennan–Prediger coefficient.

We reiterate that the agreement coefficient $\text {AC}_{1}$(Gwet, 2008) is justified using a guessing model similar to the first Maxwell’s 1977 (discussed on p. 3), but it is not a knowledge coefficient. The relationship between the $\text {AC}_{1}$ and the knowledge coefficient will be explored in a future paper.

We have only discussed a rather peculiar sort of estimation of the knowledge coefficient. It would, perhaps, be more natural to discuss traditional estimation methods, such maximum likelihood estimation, as explored by Aickin (1990) and Klauer and Batchelder (1996) in their submodels of the guessing model. In particular, composite maximum likelihood estimation (Varin et al., 2011) appears to be a good fit to the problem. Bayesian estimation could also be a reasonable option. If we only care about performance measures such as the mean squared error, the Brennan–Prediger coefficient has small bias under many scenarios, and its variance is virtually guaranteed to be smaller than the variance of a composite maximum likelihood estimator. But if we care about inference, even the superior confidence intervals for the Brennan–Prediger coefficient have unacceptably poor coverage under some circumstances. Since constructing approximate confidence intervals for maximum likelihood and composite maximum likelihood is routine, going this route will likely fix the coverage problem.

### Supplementary Information

Below is the link to the electronic supplementary material.

Supplementary file 1 (pdf 192 KB)

## Appendix

### Proof of the Knowledge Coefficient Theorem on p. 6.

Recall that *W* is a matrix $C\times C$ matrix of agreement weights, that is, a symmetric matrix whose elements $W_{ir}$ satisfy $1\ge W_{ir}$ with $W_{ir}=1$ if and only if $i=r$. We simplify our notation by considering two judges only. Define $p_{wa}(r_{1},r_{2})$ and $p_{wc}(r_{1},r_{2})$ as the the probability of (chance) agreement restricted to the two judges $r_{1}$ and $r_{2}$. Clearly, $$p_{wa}=\left( {\begin{array}{c}R\\ 2\end{array}}\right) ^{-1}\sum _{r_{1}<r_{2}}p_{wa}(r_{1},r_{2})$$ and $$p_{wc}=\left( {\begin{array}{c}R\\ 2\end{array}}\right) ^{-1}\sum _{r_{1}<r_{2}}p_{wc}(r_{1},r_{2})$$.

In the following lemma, we will view probability mass functions as vectors. That is, we will view e.g. *p* as the vector in ${\mathbb {R}}^{C}$ whose *i*th element is $p(x_{i})$. This will greatly simplify our notation.

#### Lemma 7

The following is true.

(i) The weighted agreement between $r_{1}$ and $r_{2}$, $p_{wa}(r_{1},r_{2})$, equals
  5.1 $$\begin{aligned} E[s_{r_{1}}s_{r_{2}}]+(E[s_{r_{1}}]-E[s_{r_{1}}s_{r_{2}}])t^{T}Wq_{r_{2}}+(E[s_{r_{2}}]-E[s_{r_{1}}s_{r_{2}}])q_{r_{1}}^{T}Wt\nonumber \\ +(1-E[s_{r_{1}}]-E[s_{r_{2}}]+E[s_{r_{1}}s_{r_{2}}])q_{r_{1}}^{T}Wq_{r_{2}}. \end{aligned}$$
(ii) The weighted chance agreement between $r_{1}$ and $r_{2}$, or $p_{wc}(r_{1},r_{2})$, equals
  5.2 $$\begin{aligned} E[s_{r_{1}}]E[s_{r_{2}}]t^{T}Wt+(E[s_{i_{1}r_{1}}]-E[s_{r_{1}}]E[s_{r_{2}}])t^{T}Wq_{r_{2}}+(E[s_{r_{2}}]-E[s_{r_{1}}]E[s_{r_{2}}])q_{r_{1}}^{T}Wt\nonumber \\ +(1-E[s_{r_{1}}]-E[s_{r_{2}}]+E[s_{r_{1}}]E[s_{r_{2}}])q_{r_{1}}^{T}Wq_{r_{2}}. \nonumber \\ \end{aligned}$$
(iii) Finally, the weighted chance agreement between $r_{1}$ and $r_{2}$ can be written in terms of the marginal distributions and the weighting matrix,
  5.3 $$\begin{aligned} p_{wc}(r_{1},r_{2})=\sum _{x_{1},x_{2}}w(x_{1},x_{2})p_{r_{1}}(x_{1})p_{r_{2}}(x_{2})=p_{r_{1}}^{T}Wp_{r_{2}}. \end{aligned}$$

#### Proof

**(i)** We will make use the following expression for $p_{wa}(r_{1},r_{2})$:

$$\begin{aligned} p_{wa}(r_{1},r_{2})=\sum _{x^{\star }}\sum _{x_{1}}\sum _{x_{2}}w(x_{1},x_{2})p(x_{r_{1}}\mid s,x^{\star })p(x_{r_{2}}\mid s,x^{\star })t(x^{\star }). \end{aligned}$$

Recall that $x^{\star }$ is the true rating, $x_{1}$ is the rating by judge 1, and $x_{2}$ the rating by judge 2, and the expression for the full guessing model, $p(x\mid s,x^{\star })=s_{r}1[x=x^{\star }]+(1-s_{r})q_{r}(x)$ of formula (1.1). We expand the right hand side of the expression for $p_{wa}(r_{1},r_{2})$ above to obtain

$$\begin{aligned} p_{wa}(r_{1},r_{2})&=\sum _{x^{\star }}\sum _{x_{1}}\sum _{x_{2}}w(x_{1},x_{2})s_{r_{1}}s_{r_{2}}t(x^{\star })1[x_{1}=x_{2}=x^{\star }]&(=A)\\&+\sum _{x^{\star }}\sum _{x_{1}}\sum _{x_{2}}w(x_{1},x_{2})s_{r_{1}}(1-s_{r_{2}})t(x^{\star })1[x_{1}=x^{\star }]q_{r_{2}}(x_{2})&(=B)\\&+\sum _{x^{\star }}\sum _{x_{1}}\sum _{x_{2}}w(x_{1},x_{2})s_{r_{2}}(1-s_{r_{2}})q_{r_{1}}(x_{1})t(x^{\star })1[x_{2}=x^{\star }]&(=C)\\&+\sum _{x^{\star }}\sum _{x_{1}}\sum _{x_{2}}w(x_{1},x_{2})(1-s_{r_{1}})(1-s_{r_{2}})q_{r_{1}}(x_{1})q_{r_{2}}(x_{2})t(x^{\star })&(=D) \end{aligned}$$

Now we sum over $x^{\star },x_{1},x_{2}$ for each of $(A)-(D)$. Starting with (*A*), recall that $w(x,x)=1$ for all *x*. Thus

$$\begin{aligned} A= & {} \sum _{x_{1}}\sum _{x_{2}}w(x_{1},x_{2})[s_{r_{1}}s_{r_{2}}t(x^{\star })1[x_{1}=x_{2}=x^{\star }],\\= & {} s_{r_{1}}s_{r_{2}}. \end{aligned}$$

Now consider (*B*), where we must recall that *W*, the weighting matrix, is the matrix with elements $W_{ir}=w(x_{i},x_{r}$).

$$\begin{aligned} B= & {} \sum _{x^{\star }}\sum _{x_{1}}\sum _{x_{2}}w(x_{1},x_{2})s_{r_{1}}(1-s_{r_{2}})t(x^{\star })1[x_{1}=x^{\star }]q_{r_{2}}(x_{2}),\\= & {} \sum _{x_{1}}\sum _{x_{2}}w(x_{1},x_{2})s_{r_{1}}(1-s_{r_{2}})t(x_{1})q_{r_{2}}(x_{2}),\\= & {} s_{r_{1}}(1-s_{r_{2}})t^{T}Wq_{r_{2}}. \end{aligned}$$

Likewise, $$C=s_{r_{2}}(1-s_{r_{2}})q_{r_{1}}^{T}Wt$$ and $$D=(1-s_{r_{1}})(1-s_{r_{2}})q_{r_{1}}^{T}Wq_{r_{2}}$$.

Summing over $x_{1},x_{2}$, the expression becomes

$$\begin{aligned}= & {} s_{r_{1}}s_{r_{2}}+s_{r_{1}}(1-s_{r_{2}})q_{r_{1}}^{T}Wt+s_{r_{2}}(1-s_{r_{2}})q_{r_{1}}(x_{1})t(x_{2})\\{} & {} +(1-s_{r_{1}})(1-s_{r_{2}})q_{r_{1}}^{T}Wq_{r_{2}}, \end{aligned}$$

and 5.1 follows from taking expectations over $s_{r_{1}},s_{r_{2}}$.

**(ii)** We proceed in the same way as we did in (i).

$$\begin{aligned} p_{wc}(r_{1},r_{2}\mid s_{r_{1}},s_{r_{2}})= & {} \sum _{x_{1},x_{2}}w(x_{r_{1}},x_{r_{2}})p(x_{r_{1}}\mid s_{r_{1}})p(x_{r_{2}}\mid s_{r_{1}})\\= & {} \sum _{x_{1}^{\star }}\sum _{x_{2}^{\star }}\sum _{x_{1},x_{2}}w(x_{r_{1}},x_{r_{2}})p(x_{r_{1}}\mid s_{r_{1}},x_{1}^{\star })p(x_{r_{2}}\mid s_{r_{2}},x_{2}^{\star })t(x_{1}^{\star })t(x_{2}^{\star }) \end{aligned}$$

Again, we expand this expression to obtain

$$\begin{aligned} p_{wc}(r_{1},r_{2}\mid s_{r_{1}},s_{r_{2}})&=\sum _{x_{1}^{\star }}\sum _{x_{2}^{\star }}\sum _{x_{1}}\sum _{x_{2}}w(x_{1},x_{2})s_{r_{1}}s'_{r_{2}}t(x_{1}^{\star })t(x_{2}^{\star })1[x_{1}=x_{1}^{\star }]1[x_{2}=x_{2}^{\star }]&(=A)\\&+\sum _{x_{1}^{\star }}\sum _{x_{2}^{\star }}\sum _{x_{1}}\sum _{x_{2}}w(x_{1},x_{2})s_{r_{1}}(1-s{}_{r_{2}})t(x_{1}^{\star })1[x_{1}=x^{\star }]q_{r_{2}}(x_{2})t(x_{2}^{\star })&(=B)\\&+\sum _{x_{1}^{\star }}\sum _{x_{2}^{\star }}\sum _{x_{1}}\sum _{x_{2}}w(x_{1},x_{2})s_{r_{2}}(1-s{}_{r_{1}})q_{r_{1}}(x_{1})t(x_{2}^{\star })1[x_{2}=x^{\star }]t(x_{1}^{\star })&(=C)\\&+\sum _{x_{1}^{\star }}\sum _{x_{2}^{\star }}\sum _{x_{1}}\sum _{x_{2}}w(x_{1},x_{2})(1-s_{r_{1}})(1-s{}_{r_{2}})q_{r_{1}}(x_{1})q_{r_{2}}(x_{2})t(x_{1}^{\star })t(x_{2}^{\star })&(=D) \end{aligned}$$

The main difference from (i) is in (A),

$$\begin{aligned}{} & {} \sum _{x_{1}^{\star }}\sum _{x_{2}^{\star }}\sum _{x_{1}}\sum _{x_{2}}w(x_{1},x_{2})s_{r_{1}}s{}_{r_{2}}t(x_{1}^{\star })t(x_{2}^{\star })1[x_{1}=x_{1}^{\star }]1[x_{2}=x_{2}^{\star }]\\= & {} s_{r_{1}}s'_{r_{2}}t^{T}Wt. \end{aligned}$$

Let’s consider (B) too:

$$\begin{aligned}{} & {} \sum _{x_{1}^{\star }}\sum _{x_{2}^{\star }}\sum _{x_{1}}\sum _{x_{2}}w(x_{1},x_{2})s_{r_{1}}(1-s{}_{r_{2}})t(x_{1}^{\star })1[x_{1}=x^{\star }]q_{r_{2}}(x_{2})t(x_{2}^{\star }),\\= & {} \sum _{x_{1}}\sum _{x_{2}}w(x_{1},x_{2})s_{r_{1}}(1-s{}_{r_{2}})t(x_{1})q_{r_{2}}(x_{2}),\\= & {} s_{r_{1}}(1-s_{r_{2}})t^{T}Wq_{r_{2}}. \end{aligned}$$

(C) and (D) can be calculated in the same way. After taking expectations with respect to independent $s_{r_{1}},s_{r_{2}}$, we find the expression for $p_{wc}(r_{1},r_{2})$ in the statement of the Lemma.

**(iii)** The expression for $p_{wc}$ in terms of $p_{r_{1}},p_{r_{2}}$is trivial. $\square$

#### Proof

(Proof of Theorem 2)

**(i).** Assume all guessing distributions are equal, i.e., $q_{r}(x)=q(x)$. We wish to show that the following are equivalent

5.4 $$\begin{aligned} q(x)=t(x),\quad p(x)=t(x),\quad q(x)=p(x). \end{aligned}$$

To do this, recall the marginal univariate model $p(x\mid s)=s_{r}t(x)+(1-s_{r})q(x).$ Take expectations over *s* to obtain, with $\alpha =R^{-1}\sum _{r}E(s_{r}),$

5.5 $$\begin{aligned} p(x)=\alpha t(x)+(1-\alpha )q(x). \end{aligned}$$

It immediately follows that the expressions in 5.4 are equivalent.

Let’s proceed to prove the rest of (i). If all guessing distributions are equal to the true distribution, then the formula 5.1 can be written as

$$\begin{aligned} p_{wa}(r_{1},r_{2})=E[s_{r_{1}}s_{r_{2}}]+(E[s_{r_{1}}]-E[s_{r_{1}}s_{r_{2}}])t^{T}Wt+(E[s_{r_{2}}]-E[s_{r_{1}}s_{r_{2}}])t^{T}Wt\\ +(1-E[s_{r_{1}}]-E[s_{r_{2}}]+E[s_{r_{1}}s_{r_{2}}])t^{T}Wt. \end{aligned}$$

Some of the terms cancel, leaving

$$\begin{aligned} p_{wa}(r_{1},r_{2})=E[s_{r_{1}}s_{r_{2}}](1-t^{T}Wt)+t^{T}Wt. \end{aligned}$$

Moreover, the formula for $p_{wc}(r_{1},r_{2})$ can be simplified:

$$\begin{aligned} p_{wc}(r_{1},r_{2})=E[s_{r_{1}}]E[s_{r_{2}}]t^{T}Wt+(E[s_{r_{1}}]-E[s_{r_{1}}]E[s_{r_{2}}])t^{T}Wt+(E[s_{r_{2}}]-E[s_{r_{1}}]E[s_{r_{2}}])t^{T}Wt\\ +(1-E[s_{r_{1}}]-E[s_{r_{2}}]+E[s_{r_{1}}]E[s_{r_{2}}])t^{T}Wt. \end{aligned}$$

Most of the terms cancel, leaving only

$$\begin{aligned} p_{wc}(r_{1},r_{2})=t^{T}Wt. \end{aligned}$$

To verify these formulas, simply replace all instances of $q_{r}$ with *t* in Lemma 7, parts (i) and (ii). Since the marginal distribution for every judge is the same under the assumption that $q(x)=t(x)$, it follows that $p_{wa}=p_{wa}$. Since the true distribution equals the marginal distribution,

$$\begin{aligned} t^{T}Wt=p^{T}Wp=(R^{-1}\sum _{r}p_{r})^{T}W(R^{-1}\sum _{r}p_{r})=R^{-2}\sum _{r_{1},r_{2}}p_{r_{1}}^{T}Wp_{r_{2}}, \end{aligned}$$

and by part (iii) of Lemma 7, $p_{wf}=R^{-2}\sum _{r_{1},r_{2}}p_{r_{1}}^{T}Wp_{r_{2}}$, Take the mean over all combinations of judges and reorder to arrive at

$$\begin{aligned} \nu =\frac{p_{wa}-p_{wc}}{1-t^{T}Wt}=\frac{p_{wa}-p_{wc}}{1-p_{wc}}=\frac{p_{wa}-p_{wf}}{1-p_{wf}}=\frac{p_{wa}-p_{wf}}{1-p_{wc}}. \end{aligned}$$

**(ii).** Assume that all guessing distributions are uniform. Then if $q_{r}=C^{-1}\textbf{1}$, which implies that $q_{r}^{T}p=C^{-1}$ for any probability mass function *p*. (This happens since *p* sums to 1.) It follows that $q_{r_{2}}^{T}t=q_{r_{1}}^{T}q_{r_{2}}=C^{-1}$ for all $r_{1},r_{2}$. Using expression (i) of the Lemma and $W=I$, we find that

$$\begin{aligned} p_{a}(r_{1},r_{2})=E[s_{r_{1}}s_{r_{2}}]+(E[s_{r_{1}}]-E[s_{r_{1}}s_{r_{2}}])C^{-1}+(E[s_{r_{2}}]-E[s_{r_{1}}s_{r_{2}}])C^{-1}\\ +(1-E[s_{r_{1}}]-E[s_{r_{2}}]+E[s_{r_{1}}s_{r_{2}}])C^{-1}. \end{aligned}$$

Canceling terms, we find that $p_{a}(r_{1},r_{2})=E[s_{r_{1}}s_{r_{2}}](1-C^{-1})+C^{-1}$. It follows that $\nu =\frac{p_{a}-C^{-1}}{1-C^{-1}}$.

**(iii).** Suppose that $E[s_{r_{1}}s_{r_{2}}]=E[s_{r_{1}}]E[s_{r_{2}}]$ for all $r_{1},r_{2}$. Now subtract $p_{wc}(r_{1},r_{2})$ from $p_{wa}(r_{1},r_{2})$, using Lemma 7, parts (i) and (ii). Most of the terms cancel, leaving us with

$$\begin{aligned} p_{wa}(r_{1},r_{2})-p_{wc}(r_{1},r_{2})=E[s_{r_{1}}s_{r_{2}}](1-t^{T}Wt). \end{aligned}$$

Take the mean over all combinations of judges and reorder to arrive at

$$\begin{aligned} \nu =\frac{p_{wa}-p_{wc}}{1-t^{T}Wt}. \end{aligned}$$

If the true distribution equals the marginal distribution, then $p_{wf}=p^{T}Wp=t^{T}Wt$, as explained in (i), hence $\nu =\frac{p_{wa}-p_{wc}}{1-p_{wf}}$, as claimed. On the other hand, if the true distribution is uniform, $t=C^{-1}\textbf{1}$, hence $\nu =\frac{p_{wa}-p_{wc}}{1-\textbf{1}^{T}W\textbf{1}/C^{-2}}$. $\square$

### Proof of the Expression for $\sigma _{CF}$ in Proposition 5

First, let us recall the multidimensional delta method. Let $f:{\mathbb {R}}^{k}\rightarrow {\mathbb {R}}$ be continuously differentiable at $\theta$ and suppose that $\sqrt{n}({\hat{\theta }}-\theta ){\mathop {\rightarrow }\limits ^{d}}N(0,\Sigma )$. Then

5.6 $$\begin{aligned} \sqrt{n}[f({\hat{\theta }})-f(\theta )]{\mathop {\rightarrow }\limits ^{d}}N(0,\nabla f(\theta )^{T}\Sigma \nabla f(\theta )) \end{aligned}$$

In the case of the Cohen–Fleiss coefficient, $\theta =(p_{wa},p_{wc},p_{wf})$ and $f(\theta )=\frac{p_{wa}-p_{wc}}{1-p_{wf}}$. Then

$$\begin{aligned} \nabla f=\frac{1}{1-p_{wf}}\left( 1,-1,\nu _{CBP}\right) . \end{aligned}$$

$$\begin{aligned} (p_{wa},p_{wc},p_{wf}) \end{aligned}$$

Thus the variance is

$$\begin{aligned} (1-p_{wf})^{2}\nabla f(\theta )^{T}\Sigma \nabla f(\theta )= & {} \left( 1,-1,\nu _{CBP}\right) ^{T}\Sigma \left( 1,-1,\nu _{CBP}\right) ,\\= & {} p_{wa}\sigma _{wa}^{2}+p_{wc}^{2}\sigma _{wc}^{2}+p_{wf}^{2}\sigma _{wf}^{2}+2\sigma _{wf}^{2}\sigma _{wc}^{2}. \end{aligned}$$

### Proof that Aickin’s Model is a Guessing Model

#### Lemma 8

Let the number of judges be 2 and $s\sim \text {Bernoulli}(\alpha )$ be the same for both judges. Then the guessing model has unconditional distribution

5.7 $$\begin{aligned} p(x_{1},x_{2})=\alpha 1[x_{1}=x_{2}]t(x_{1})+(1-\alpha )q_{1}(x_{1})q_{2}(x_{2}). \end{aligned}$$

#### Proof

Expanding the guessing model 1.1

$$\begin{aligned} p(x_{1},x_{2}\mid s,x^{\star })= & {} s^{2}1[x_{1}=x^{\star }]1[x_{2}=x^{\star }]\\= & {} +s(1-s)1[x_{1}=x^{\star }]q_{2}(x_{2})\\{} & {} +s(1-s)1[x_{2}=x^{\star }]q_{1}(x_{1})\\{} & {} +(1-s)(1-s)q_{1}(x_{1})q_{2}(x_{2}) \end{aligned}$$

Since $s\sim \text {Bernoulli}(\alpha )$, we have that $Es^{2}=\alpha$, $E(s(1-s))=0$ and $E(1-s)(1-s)=1-\alpha$. It follows that

$$\begin{aligned} p(x_{1},x_{2}\mid x^{\star })=\alpha 1[x_{1}=x^{\star }]1[x_{2}=x^{\star }]+(1-\alpha )q_{1}(x_{1})q_{2}(x_{2}). \end{aligned}$$

Summing over $x^{\star }$ yields $p(x_{1},x_{2})=\alpha 1[x_{1}=x_{2}]t(x_{1})+(1-\alpha )q_{1}(x_{1})q_{2}(x_{2})$. $\square$
