# Supplementary material for: Measuring Agreement Using Guessing Models and Knowledge Coefficients
Source: Psychometrika. 2023 Jun 8;88(3):1002–25. doi: 10.1007/s11336-023-09919-4 (PMC10444669; doi:10.1007/s11336-023-09919-4)
Supplement: Supplementary file 1 — (pdf 192 KB) [file 11336_2023_9919_MOESM1_ESM.pdf]

Online appendix

More sensitivity analysis

This is a continuation of the sensitivity analysis of section 10 when $\rho = 0.5$ and $\rho = 0.9$. If you compare these Tables with Tables 4 (p. on page 12) and 5 (p. on page 12) you will find few differences.

Table 9: Sensitivity analysis. True distribution centered on the uniform distribution ($\rho = 0.9$).

Variability		Coefficient				
Guessing*	True*	Cohen-Fleiss	Fleiss	Cohen	BP	Cohen–BP
	None	**0.0e+00**	**0.0e+00**	**0.0e+00**	1.1e-02	2.2e-02
None	Low	**2.2e-02**	**2.2e-02**	**2.2e-02**	**1.2e-02**	**8.3e-02**
	High	3.3e-01	3.3e-01	3.3e-01	**1.8e-02**	4.6e-01
	None	**2.8e-05**	3.4e-04	2.4e-04	1.4e-02	3.1e-02
Low	Low	**2.3e-02**	**2.3e-02**	**2.3e-02**	**1.4e-02**	**9.0e-02**
	High	3.4e-01	3.4e-01	3.4e-01	**2.2e-02**	4.7e-01
	None	**2.3e-04**	2.8e-03	1.9e-03	3.1e-02	7.1e-02
High	Low	**2.4e-02**	**2.7e-02**	**2.6e-02**	**3.1e-02**	1.3e-01
	High	3.4e-01	3.5e-01	3.4e-01	**3.7e-02**	4.7e-01

* Variability of the true distributions: Baseline: True distribution is uniform.
* Variability of the guessing distributions. Baseline: All guessing distributions are equal to the true distribution.

Table 10: Sensitivity analysis. True distribution centered on the marginal guessing distribution ($\rho = 0.9$).

Variability		Coefficient				
Guessing*	True*	Cohen–Fleiss	Fleiss	Cohen	BP	Cohen–BP
	None	**0.0e+00**	**0.0e+00**	**0.0e+00**	**0.0e+00**	**0.0e+00**
None	Low	3.9e-03	4.0e-03	3.9e-03	**0.0e+00**	1.2e-02
	High	7.7e-02	7.8e-02	7.7e-02	**0.0e+00**	1.7e-01
	None	5.5e-05	4.4e-05	2.8e-05	4.4e-05	**0.0e+00**
Low	Low	3.7e-03	3.8e-03	3.8e-03	**7.3e-04**	1.2e-02
	High	7.5e-02	7.6e-02	7.5e-02	**2.5e-03**	1.7e-01
	None	8.3e-04	5.9e-04	4.0e-04	6.3e-04	**0.0e+00**
High	Low	**3.6e-03**	**4.5e-03**	**4.1e-03**	**2.8e-03**	1.2e-02
	High	**7.4e-02**	**7.6e-02**	**7.5e-02**	**1.1e-02**	1.7e-01

* Variability of the true distributions: Baseline: True distribution equals the marginal guessing distribution.
* Variability of the guessing distributions. Baseline: All guessing distributions are equal.

Exact variance of $\hat{p}_a$ and $\hat{\nu}_{BP}$

In this section we derive the exact, non-asymptotic variance of $\hat{p}_a$ and the Brennan–Prediger coefficient $\hat{\nu}_{BP}$.

Proposition 9. *The variance of*

$$\hat{p}_a = n^{-1} \sum_{i=1}^{n} \binom{R}{2}^{-1} \sum_{r>r'} 1[X_{ir} = X_{ir'}]$$

is $\operatorname{Var}\hat{p}_a = n^{-1}(p_{2a} - p_a^2)$. *Here*

$$p_{2a} = \binom{R}{2}^{-2} \sum_{r_1>r_1'} \sum_{r_2>r_2'} \mu_{r_1 r_1' r_2 r_2'},$$

and

$$\mu_{r_1 r_1' r_2 r_2'} = P(X_{ir_1} = X_{ir_1'}, X_{ir_2} = X_{ir_2'}).$$

Table 11: Sensitivity analysis. True distribution centered on the uniform distribution ($\rho = 0.5$).

Variability		Coefficient				
Guessing⋆	True*	Cohen–Fleiss	Fleiss	Cohen	BP	Cohen–BP
None	None	**0.0e+00**	**0.0e+00**	**0.0e+00**	1.1e-02	2.3e-02
	Low	**2.2e-02**	**2.2e-02**	**2.2e-02**	**1.2e-02**	**8.2e-02**
	High	3.4e-01	3.4e-01	3.4e-01	**1.9e-02**	4.6e-01
Low	None	**0.0e+00**	3.9e-04	2.5e-04	1.5e-02	3.2e-02
	Low	**2.3e-02**	**2.3e-02**	**2.3e-02**	**1.6e-02**	**9.2e-02**
	High	3.3e-01	3.4e-01	3.4e-01	**2.3e-02**	4.6e-01
High	None	**0.0e+00**	3.0e-03	1.9e-03	3.8e-02	8.8e-02
	Low	**2.4e-02**	**2.7e-02**	**2.6e-02**	**4.0e-02**	1.4e-01
	High	3.4e-01	3.5e-01	3.4e-01	**4.7e-02**	4.7e-01

* Variability of the true distributions: Baseline: True distribution is uniform.
⋆ Variability of the guessing distributions. Baseline: All guessing distributions are equal to the true distribution.

Table 12: Sensitivity analysis. True distribution centered on the marginal guessing distribution ($\rho = 0.5$).

Variability		Coefficient				
Guessing⋆	True*	Cohen–Fleiss	Fleiss	Cohen	BP	Cohen–BP
None	None	**0.0e+00**	**0.0e+00**	**0.0e+00**	6.60e-02	1.44e-01
	Low	2.19e-01	2.19e-01	2.19e-01	**4.89e-02**	3.24e-01
	High	5.95e-01	5.95e-01	5.95e-01	**4.88e-02**	6.33e-01
Low	None	**7.30e-05**	3.81e-04	2.84e-04	4.90e-02	1.08e-01
	Low	**2.99e-02**	**3.02e-02**	**3.01e-02**	**5.05e-02**	1.65e-01
	High	3.47e-01	3.47e-01	3.47e-01	**5.38e-02**	4.83e-01
High	None	**6.00e-04**	3.13e-03	2.33e-03	6.48e-02	1.47e-01
	Low	**3.14e-02**	**3.36e-02**	**3.28e-02**	**6.47e-02**	1.91e-01
	High	3.47e-01	3.52e-01	3.48e-01	**6.71e-02**	4.93e-01

* Variability of the true distributions: Baseline: True distribution equals the marginal guessing distribution.
⋆ Variability of the guessing distributions. Baseline: All guessing distributions are equal.

Proof. Define $\mu_{rr}' = P(X_{ir} = X_{ir'})$. Then

$$p_a = \binom{R}{2}^{-1} \sum_{r>r'} P(X_{ir} = X_{ir'}) = \binom{R}{2}^{-1} \sum_{r>r'} \mu_{rr}'$$

by definition and thus,

$$p_a^2 = \left[\binom{R}{2}^{-1} \sum_{r>r'} \mu_{rr}'\right]^2 = \binom{R}{2}^{-2} \sum_{r_1>r_1'} \sum_{r_2>r_2'} \mu_{r_1 r_1'} \mu_{r_2 r_2'}.$$

To find the variance we must calculate

$$\begin{aligned} E\hat{p}_a^2 &= n^{-2}\binom{R}{2}^{-2}\sum_{i_1,i_2}\sum_{r_1>r_1'}\sum_{r_2>r_2'} E\left\{1[X_{i_1r_1}=X_{i_1r_1'}]1[X_{i_2r_2}=X_{i_2r_2'}]\right\}, \\ &= n^{-2}\binom{R}{2}^{-2}\sum_{i_1,i_2}\sum_{r_1>r_1'}\sum_{r_2>r_2'} P(X_{i_1r_1}=X_{i_1r_1'},X_{i_2r_2}=X_{i_2r_2'}). \end{aligned}$$

Looking at $P(X_{i_1r_1} = X_{i_1r_1'}, X_{i_2r_2} = X_{i_2r_2'})$, we notice that, if $i_1 \neq i_2$ it equals $P(X_{i_1r_1} = X_{i_1r_1'}, X_{i_2r_2} = X_{i_2r_2'}) = \mu_{r_1r_1'}\mu_{r_2r_2'}$ due to independence. This happens $n(n-1)$ times when we sum over i_1, i_2. On the other hand, if $i_1 = i_2$, it equals $\mu_{r_1r_1'r_2r_2'}$. This happens n times when we sum over i_1, i_2.

It follows that

$$E\hat{p}_a^2 = (1-n^{-1})p_a^2 + n^{-1}\binom{R}{2}^{-2}\sum_{r_1>r_1'}\sum_{r_2>r_2'}\mu_{r_1r_1'r_2r_2'},$$

and the variance equals

$$\operatorname{Var}\hat{p}_a = n^{-1}(E\hat{p}_a^2 - p_a^2) = n^{-1}(p_{2a} - p_a^2),$$

as claimed. □

The sample Brennan–Prediger coefficient $\hat{\nu}_{BP} = (\hat{p}_a - C^{-1})/(1 - C^{-1})$ is easy to deal with, as it's a linear function of $\hat{p}_a$. As shown in Proposition 23, the sample agreement $\hat{p}_a$ has exact variance $\operatorname{Var}(\hat{p}_a) = n^{-1}(p_{2a} - p_a^2)$, where p_a is the true agreement and$p_{2a} = \binom{R}{2}^{-2}\sum_{r_1>r_1'}\sum_{r_2>r_2'} P(X_{ir_1} = X_{ir_1'}, X_{ir_2} = X_{ir_2'})$. The value of p_{2a} is easy to estimate using the sample estimator $\hat{p}_{2a}$. Moreover, the expectation of $\hat{p}_a^2$ is $\frac{n-1}{n}p_a^2 + \frac{1}{n}p_{2a}$, hence a debiased estimator of $n^{-1}(p_{2a} - p_a^2)$ is

$$\hat{\operatorname{Var}}\hat{p}_a = (n-1)^{-1}(\hat{p}_{2a} - \hat{p}_a^2),$$

provided $\hat{p}_{2a} > \hat{p}_a^2$. This inequality is rarely broken, even in small samples.

Using the expression for $\operatorname{Var}\hat{p}_a$, it follows that the exact variance of the estimated Brennan–Prediger coefficient $\hat{\nu}$ is

$$\operatorname{Var}\hat{\nu} = n^{-1}(p_{2a} - p_a^2)\frac{C^2}{(C-1)^2}, \tag{6.8}$$

and $\sqrt{n}(\hat{\nu} - \nu)$ is asymptotically normal by the delta method. A natural plug-in estimator of $\operatorname{Var}\hat{\nu}$ is then

$$\hat{\operatorname{Var}}\hat{\nu} = (n-1)^{-1}(\hat{p}_{2a} - \hat{p}_a^2)\frac{C^2}{(C-1)^2}$$

The usual estimator of $\operatorname{Var}\hat{p}_a$ is $n^{-1}(n-1)^{-1}\sum(\hat{p}_a(i) - \hat{p}_a)^2$, where $\hat{p}_a(i)$ is the probability of agreement restricted to the ith item. This estimator is consistent since $\hat{p}_a = \frac{1}{n}\sum_{i=1}^{n}\hat{p}_a(i)$. We would recommend this estimator of the variance over the plug-in estimator for three reasons. First, it performs better than the plugin estimator in several scenarios. Second, it is always non-negative, being zero if and only if all $\hat{p}_{ai}$s are equal. Finally, it is considerably less computationally expensive.
